# Fluorescein-guided surgical ablation of spinal dural arteriovenous fistula (SDAVF) is a safe and effective add-on to ensure surgical closure

**DOI:** 10.1016/j.bas.2026.105978

**Published:** 2026-02-12

**Authors:** Seyar Entezari, Mathias Møller Thygesen, Gudrun Gudmundsdottir, Jakob Gram Carlsen, Ronni Mikkelsen, Mikkel Mylius Rasmussen

**Affiliations:** a Department of Neurosurgery, Aarhus University Hospital, Aarhus, Denmark; b Cense-Spine, Aarhus University Hospital, Aarhus, Denmark; c Department of Radiology section of Neuroradiology, Aarhus University Hospital, Aarhus, Denmark; d Department of clinical medicine, Aarhus University, Aarhus, Denmark

**Keywords:** Spinal dural arteriovenous fistula (SDAVF), Spinal arteriovenous lesions (SAVLs), T2 high-signal intensity area, intramedullary edema

## Abstract

**Introduction:**

Spinal dural arteriovenous fistula (SDAVF) is the most common subtype of spinal arteriovenous lesions. SDAVF often causes intramedullary edema, resulting in ischemic myelopathy and progressive paraplegia over time. Identifying the SDAVF intraoperatively is not always easy. Fluorescein-guided surgical ablation is a technique developed in our department to visualize the fistula in SDAVF enabling secure surgical closure.

**Research question:**

To evaluate the efficiency of fluorescein-guided open surgical ligation treatment of SDAVFs.

**Material and Methods:**

Single centre retrospective cohort study.

**Results:**

22 patients (male = 18), 34 - 83 years of age were included. Patients presented with gait disturbance (n=22) balance disturbances (n=20), back pain (n=15), and/or radicular pain to the lower extremities (n=13). Fluorescein-guided surgical ablation was applied peri-operatively in all patients. Successful identification of SDAVF was seen in all patients. One had re-surgery; in this patient the fluorescein-guided was not followed and dural leakage was observed. No complication to the add-on procedure was seen. After treatment, 19 patients experienced either complete remission- or some degree of improvement in gait disturbance (RR=0.86 (95% Confidence interval (CI) 0.65-0.97)), balance disturbances ((RR=0.85 (95%CI 0.62-0.97)), back pain ((RR=0.53 (95%CI 0.27-0.79)), and/or radicular pain ((RR=0.69 (95%CI 0.39-0.91)). Rostro-caudal extent of intramedullary edema on Magnetic resonance imaging was reduced by a mean 161 mm (95%CI 129-194) postoperatively.

**Discussion and Conclusion:**

Fluorescein-guided open surgical ablation of SDAVF is safe and seems a low cost effective diagnostic add-on with postoperative clinical- and imaging improvement.

## Highlights

• Fluorescein allows real-time visualization of Spinal Dural Arteriovenous Fistulas
• Fluorescein confirms complete closure of Spinal Dural Arteriovenous Fistulas
• Fluorescein-guided Spinal Dural Arteriovenous Fistulas surgery improves outcomes
• Fluorescein-guided Spinal Dural Arteriovenous Fistulas surgery is safe and low-cost

## Introduction

Spinal dural arteriovenous fistula (SDAVF) is the most common subtype of spinal arteriovenous lesions, representing 80–85 % of spinal cord vascular shunts (Sundbye et al., 2018; Flores et al., 2017). SDAVF has been hypothesized to be caused by infection, syringomyelia, trauma or surgery. The incidence is estimated around 5–10 per million, and predominantly affects men with a mean age of 60 years (Sundbye et al., 2018; McKinley et al., 2007; Zanin et al., 2024; El Naamani et al., 2024). SDAVF is a low-flow shunt between a radicular artery and peri-medullary veins causing venous stasis followed by intramedullary edema and ischemic myelopathy (Gobin et al., 1992; Hacein-Bey et al., 2014; Horikoshi et al., 2000; Jellema et al., 2003; Jeng et al., 2015; Rathnam and Memon, 2020; Kiyosue et al., 2017). Symptoms are often non-specific signs of progressive myelopathy, including gait- and balance disturbance, back pain with- or without radicular distribution, spasticity, and motor and sensory deficits (Hacein-Bey et al., 2014; Saladino et al., 2010). SDVAF predominantly occurs in the thoracolumbar region (Rathnam and Memon, 2020; Kiyosue et al., 2017; Safaee et al., 2018) and can be visualized by either Magnetic resonance imaging (MRI) (Brinjikji et al., 2016; Krings et al., 2005; Krings and Geibprasert, 2009; Morris, 2012; TaKai, 2017) or spinal digital subtraction angiography (DSA) (Krings et al., 2005; Krings and Geibprasert, 2009; Morris, 2012; Amarouche et al., 2015; Huffmann et al., 1995; Raymaekers et al., 2024). Obliteration of the draining vein can either be done by conventional open surgery or endovascular embolization (Bakker et al., 2015; Jellema et al., 2006; Mascalchi et al., 1995; Park et al., 2008; Patel et al., 2013; Ropper et al., 2012; Musmar et al., 2025; Garcia-Garcia et al., 2024; Peng et al., 2023). In both scenarios, identification of the shunt can be challenging due to the complex vascular anatomy. As a result, failed or incomplete closure is a risk (Musmar et al., 2025; Lee et al., 2021).

Inspired by the use of Fluorescein-guided surgery for cerebral arteriovenous malformations (Zhao et al., 2019; Lane et al., 2014), our centre has implemented Fluorescein-guided surgery for SDVAF aiming to improving surgical safety and closure rate. Although dynamic, the preoperative DSA may be difficult to translate into the actual surgical field. Contrary intraoperative fluorescein provides real-time hemodynamic information, complementing angiographic findings by intraoperatively demonstrating flow direction, venous hypertension patterns, and detailed feeder anatomy, as well as enabling confirmation of complete disconnection (Lane and Cohen-Gadol, 2014). We report a consecutive case cohort after introduction to evaluate the efficiency of fluorescein-guided open surgical ligation treatment of SDAVFs.

## Methods

### Patient cohort and Data collection

Patients were identified and data extracted from the hospital records at Aarhus University Hospital between January 1st 2012 and December 31st 2022. The study period ended in 2022 to ensure sufficient follow-up time for assessing long-term outcome and complication rate. Diagnosis-code (D1770B) and spinal DSA procedure-code (UXAE60) were applied as spinal DSA is a requirement prior to surgery. Patients included had to have a preoperative MRI, a postoperative MRI and/or neurological evaluation, and intraoperative use of fluorescein before and after ligation of the SDAVF.

### Surgical procedure

The surgical procedure involves ligation of the SDAVF by coagulation following a laminectomy under microscopic magnification. After the laminectomy and opening of the dura, but before ligating the suspected fistula, 5–10 mg of 10% fluorescein sodium solution is administered via a peripheral venous catheter. The dye must be delivered as a rapid intravenous bolus – injection speed of around 5 ml/seconds – through a peripheral line to ensure a sharp arterial-phase fluorescence peak. Under blue light (465–490 nm) and visualized with a dual-light microscope, the fluorescein emits a bright yellow-green fluorescence. After injection, the arterial phase typically appears at approximately 20-30 seconds, followed by venous filling at about 30-50 seconds, and tissue diffusion thereafter. The surgeon carefully observes whether the suspected vessel fills in a retrograde direction during the arterial phase, where the pathologically dilated medullary veins may show early fluorescence, while recording the sequence for later review. This retrograde arterial-phase filling helps identify the intradural point of origin—typically along a peripheral nerve—which corresponds to the arteriovenous fistula and is then prepared for ligation as close to the dura as possible. Following ligation, the surgical field is re-evaluated under blue light after administering a new dose of fluorescein. To avoid interference from residual dye, a minimum interval of five minutes is maintained between the two administrations. This second evaluation confirms complete SDAVF closure (see Fig. 1 and Supplementary Videos 1 and 2).

**Fig. 1:**
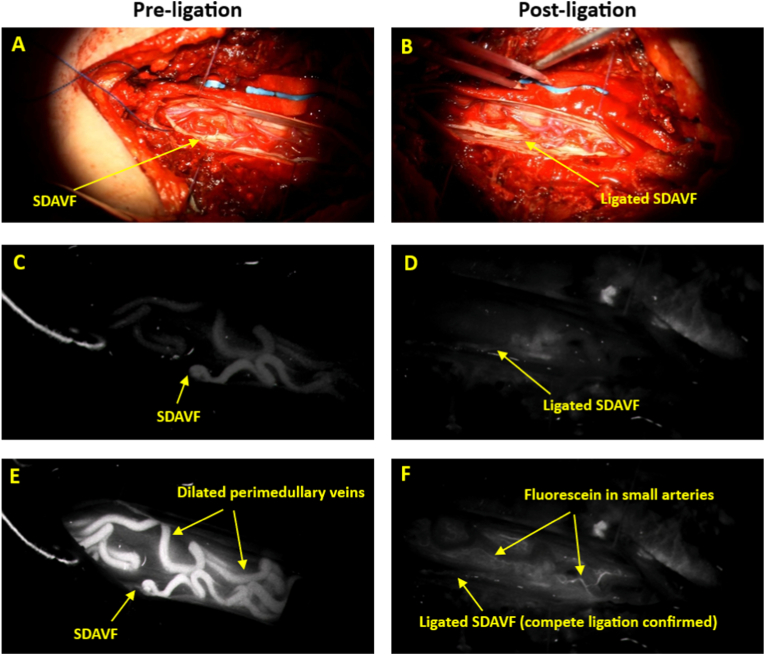
Fluorescein for SDAVF Visualization and Ligation Check. Legend Fig. 1: Key stages of surgical ligation of a spinal dural arteriovenous fistula (SDAVF), visualized under both white-light (top two images) and blue-light microscopy (bottom four images), before (left panel) and after ligation (right panel). **A)***Pre-ligation, white light:* The SDAVF is marked on the exposed spinal cord. **B)***Post-ligation, white light:* The ligation site is shown. **C)***Pre-ligation, blue light, early phase (approximately 25 seconds after fluorescein injection):* the dye reaches the SDAVF at the surgical site. **D)***Pre-ligation, blue light, late phase (approximately 40 seconds after fluorescein injection):* Fluorescein fills dilated medullary veins, indicating abnormal venous drainage due to the active fistula. **E)***Post-ligation, blue light, early phase (approximately 25 seconds after fluorescein injection):* No fluorescein is seen in the venous system, indicating successful shunt disruption. **F)***Post-ligation, blue light, late phase (approximately 40 seconds after fluorescein injection):* Small physiological arteries are fluorescent, no dilated veins, confirming complete SDAVF ligation.

### Imaging data

The rostro-caudal extent of the edema and intradural dilated and tortuous vessels on sagittal T2 weighted MRI was measured at baseline and follow-up. DSA were reviewed for the level and laterality of the SDAVF. Date of DSA was defined as the date of diagnosis.

### Statistical analysis

The statistical analysis was performed using STATA 16. Proportions including the corresponding 95% Confidence interval (CI) are presented for binary outcomes. Continuous variables are presented with mean, mean difference including the corresponding 95% CI.

### Ethics and approvals

The study was approved by the Central Denmark Region under rules and regulations from the Danish patient safety authority (no. 1-45-70-136-23).

## Results

### Patient demographic data

Demographic data are summarized in Table 1. The majority was males with an average age of 62 years of age (range 34 - 83 years of age). The level of SDAVF ranged between C1 and S1 and was left sided in 15 patients, right sided in 6 patients and bilateral in 1 patient. Symptom duration median was 12 days (range 1 - 132 days). The majority of patients received treatment within seven days after diagnosis.

**Table 1:** Baseline demographic data

Age	Sex	Symptom duration* (months)	Time to operation** (days)	Level of the SDAVF	Side of the SDAVF	AIS-baseline (follow-up)
34	Male	1	2	L1	Right	D (D)
35	Male	9	3	T6	Left	D (D)
46	Male	3	3	T12	Left	D (D)
47	Male	6	2	C1	Left	C (D)
56	Female	12	0	L3	Left	C (D)
57	Female	1	6	L3	Right	D (D)
57	Male	31	23	T12	Left	D (D)
58	Male	128	26	T8	Bilateral	D (D)
59	Male	12	18	L4	Left	D (D)
60	Male	1	0	T10	Left	D (D)
63	Male	1	3	L1	Left	D (D)
64	Male	4	7	T4	Right	D (D)
65	Male	12	3	T12	Left	D (D)
67	Male	4	1	T12	Left	D (D)
70	Male	29	1	T8	Right	D (D)
71	Male	9	7	T12	Left	D (D)
71	Male	6	1	L2	Right	C (D)
72	Female	12	6	T10	Right	D (D)
76	Male	93	2	L1	Left	D (D)
76	Male	2	3	T12	Left	D (D)
77	Male	10	0	S1	Left	D (D)
83	Female	5	0	T11	Left	D (D)

Legend Table 1: Baseline demographic data. *Symptom duration was defined as time interval between initial symptoms and diagnosis. **Time to operation was defined as time from diagnosis to surgery.

In four patients, the definitive diagnosis was done during surgery. In these patients, date of diagnose was noted as date of surgery instead of DSA date.

### Surgical results

Intraoperative fluorescein injection confirmed complete occlusion of the SDAVF in all patients. However, postoperative imaging revealed incomplete occlusion in one case, likely due to a double feeder. This patient has a dural leakage as well. Sufficient time between injections before and after ligation was not followed in this case. The procedure was done at the beginning of implementation. This would allow residual dye to obscure the second feeder and result in incomplete occlusion. The patient underwent redo surgery. No peri- or postoperative complications were registered for all patients, saving the above.

### Clinical evaluation

Clinical evaluation are summarized in Table 2. Patients predominantly presented with gait disturbance (n=22), balance disturbances (n=20), back pain (n=15), and/or radicular pain to the lower extremities (n=13). All patients had improvement in their clinical evaluation at follow-up. AIS score improved from C to D in 3 patients (see Table 1).

**Table 2:** Symptom improvement (baseline to follow-up)

	Patients improved (Follow-up improvement/baseline symptomatic) (n)
Gait disturbance	19/22
Balance disturbance	17/20
Back Pain	8/15
Radicular pain	9/13
Neurogenic bladder- or bowel dysfunction	4/15
Spasticity	3/3
Headache	1/2
Cramping	1/1

Legend Table 2: Improvement of neurological symptoms from baseline to follow-up.

### Imaging findings

MRI data at baseline and follow-up were available in 17 patients. Table 3 summarizes our findings.

**Table 3:** Imaging findings (17 patients)

Variable	Pre surgery	Follow-up	Average improvement (95% CI)	P-value
T2 hyperintensity	17/17	2/17	-	-
Multiple intradural dilated and tortuous vessels	13/17	0/17	-	-
Reduction in extent of T2 hyperintensity (mm)	170	9	161 (129; 194)	< 0.0001
Reduction in extent of multiple intradural dilated and tortuous vessels (mm)	78	0	78 (34; 122)	0.0015

Legend Table 3: Imaging findings. Baseline- and follow-up MRIs were available for 17 patients. Mean follow-up time 12.4 months ((95%CI (6.75:18.04)).

The mean rostro-caudal extent of T2 high-intensity areas was significantly reduced (mean reduction was 161 mm (95% CI: 129–194)) and a complete remission of dilated and tortuous vessels was found in all patients with baseline imaging evidence. Baseline imaging data were average 34 days (range 2- and 132 days) prior to surgery, and follow-up mean was 12.4 months (range 2- and 49 months) postoperatively.

## Discussion

Fluorescein-guided surgical ablation of the SDAVF resulted in complete ligation in all patients in our consecutive cohort except one case in the early phase of introduction, in whom postoperative imaging revealed a persisting fistula at an adjacent level. No peri- or postoperative complications occurred, except for a dural leak in that same patient. These results support open surgery as an effective SDAVF treatment, with fluorescein enhancing intraoperative visualization.

A systematic review of 18 studies involving 814 patients demonstrated that open surgery is associated with a significantly lower failure rate compared to endovascular treatment (5% vs. 46%, p < 0.01) (Zanin et al., 2024). While overall complication rates were similar, permanent neurological complications occurred more often in the endovascular group, suggesting open surgery may provide more reliable and safer long-term outcomes (Zanin et al., 2024).

However, not all studies show a clear advantage of one modality over the other. A retrospective observational study of 24 patients reported no statistically significant differences in occlusion or complication rates between surgical and endovascular approaches (El Naamani et al., 2024). Another study found similar clinical improvement with both treatments, but surgery achieved higher occlusion rates (91.3% vs. 70%) and lower recurrence rates (9.1% vs. 21.4%) (Bretonnier et al., 2019).

Supporting these trends, a meta-analysis of 40 studies reported complete occlusion rates of 96.8% for open surgery versus 72.5% for endovascular embolization, and initial treatment failure rates of 2.3% and 32.2%, respectively (Musmar et al., 2025). Notably, incomplete occlusion is more likely in patients with variant vascular anatomy and multiple arterial feeders to the shunting vein, highlighting the critical importance of accurate fistula localization for effective treatment (Lee et al., 2021; O'Reilly et al., 2022). Intraoperative fluorescein injection is intended to visualize the fistula pre-ligation, while a second post-ligation helps confirm complete closure. Once injected, fluorescein circulates through the blood vessels and gradually diffuses into the surrounding soft tissues, typically clearing after about 4–5 minutes. For optimal visualization, it is recommended to wait at least 5 minutes between the initial (pre-ligation) and the second (post-ligation) injection. Fluorescein has high membrane permeability, binds minimally to serum proteins and diffuses rapidly through soft tissue (Zhao et al., 2019; Sulaiman et al., 2015); thus, residual background fluorescence can persist in the dura and epidural fat for several minutes. This waiting interval is therefore a critical technical parameter to ensure accurate visualization. In SDAVF specifically, dilated venous plexus structures may retain fluorescein longer, meaning inadequate washout can obscure persistent venous reflux or additional feeders. We therefore recommend waiting at least 5 minutes—and up to 7 minutes in cases with large venous lakes—before administering the second injection. This interval ensures that residual dye from the first injection has cleared, reducing the risk of obscuring a small residual fistula or a second feeder. During early implementation of the fluorescein-guided surgery procedure at our institute, the recommended five-minute interval between pre- and post-ligation injections was not strictly followed. In our study, the one patient with incomplete occlusion may have had a double feeder to the shunting vein that went undetected during surgery. In this case, residual dye from the first injection may have obscured the smaller feeder, preventing its identification and ligation. Although the main shunting vein was successfully closed, persistent flow through the presumed unrecognized secondary feeder could have redirected venous pressure, promoting its enlargement and possibly predisposing to fistula recurrence. This interpretation is consistent with a previous observation in which post-ligation fluorescein administration enabled the detection of a residual fistula (Bretonnier et al., 2018). Additionally, ongoing venous hypertension from the suspected residual shunt may have increased intradural pressure, contributing to the postoperative dural leak. This case highlights several critical lessons: strict adherence to the waiting interval is essential for accurate visualization of all feeders, unrecognized secondary feeders can compromise outcomes even when the primary shunt is occluded, and postoperative dural leaks may serve as an early warning of residual shunting. Incorporating these insights into the procedure may help prevent recurrence and optimize outcomes in future cases.

Fluorescein is generally safe, with infrequent mild reactions such as nausea or vomiting, and rare serious reactions (Butner and McPherson, 1983; Karhunen et al., 1987; Kwan et al., 2006). In surgery for cerebral arteriovenous malformations, repeated 1–1.5 mg fluorescein boluses per kilogram body weight are usually administered to assess optimal fluorescent vascular imaging (Lane and Cohen-Gadol, 2014; Zhao et al., 2019), highlighting that our substantially lower doses—of 5-10 mg boluses—is sufficient for visualization while minimizing systemic risk. Our findings suggest that fluorescein-guided surgery for SDAVF is safe and could be an effective, low-cost add-on procedure, that could enhance safety by providing clear, real-time visualization of abnormal vascular structures.

Although all patients showed imaging improvement postoperatively, clinical improvement varied. This aligns with previous reports showing no consistent correlation between MRI changes and clinical outcome after SDAVF treatment (Horikoshi et al., 2000). The primary goal remains halting symptom progression (McKinley et al., 2007). Imaging improvement may indicate disease stabilization, even in the absence of full clinical recovery.

Our study found an average diagnostic delay of 24 days, which is shorter than previously reported delays of several months (Jellema et al., 2003; Brinjikji et al., 2016; Mascalchi et al., 1995; Ikeuchi et al., 2024). The nonspecific and progressive nature of SDAVF symptoms likely contributes to diagnostic challenges (Hacein-Bey et al., 2014; Saladino et al., 2010; Brinjikji et al., 2016). Earlier MRI—especially when signs of upper motor neuron involvement appear—may aid in timely diagnosis and intervention.

Radiological improvement was observed as early as two days postoperatively in our cohort, which supports prior findings that intramedullary edema can start to resolve within 1–4 months after treatment (Horikoshi et al., 2000).

This study is limited by the absence of a direct comparison with conventional ligation. Given the rarity of SDAVF, the study was designed to evaluate the effectiveness and safety of fluorescein-guided ligation rather than its comparative efficacy, though future multicentre studies could address this.

## Conclusion

Fluorescein-guided open surgical ablation of SDAVF seems to be a safe and effective procedure with postoperative clinical- and imaging improvement.

## Figures and Tables

**Figs1:**
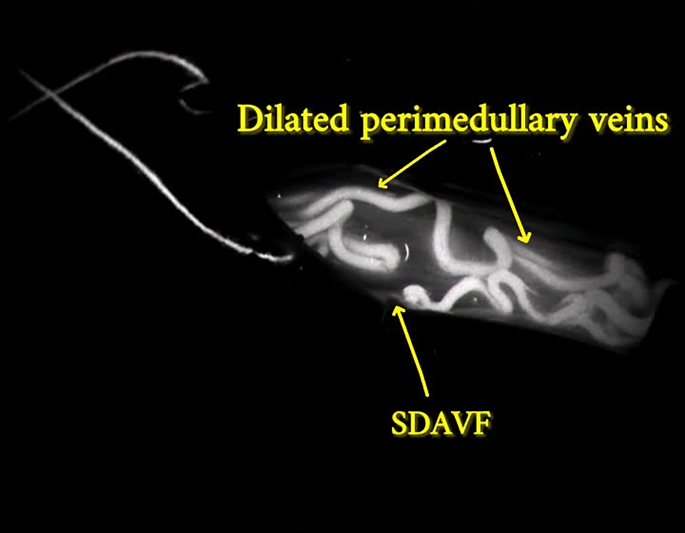


**Figs2:**
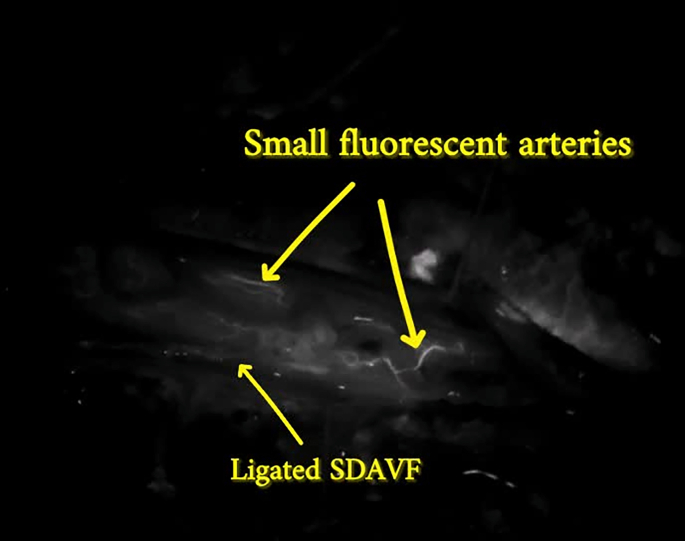


## Appendix A Supplementary data

Supplementary Video 1: Fluorescein Flow Before SDAVF Ligation Description: Following intravenous fluorescein administration, the dye rapidly flows through the shunting vein and enters the dilated medullary veins, confirming pathological venous drainage due to the SDAVF. This dynamic fluorescence highlights the abnormal vascular anatomy prior to surgical ligation. Multimedia component. 1

Supplementary Video 2**:** Fluorescein Flow After SDAVF Ligation Description: After ligation of the SDAVF, fluorescein no longer passes through the shunting vein, which is now obliterated. There is no opacification of medullary veins. Fluorescein is instead visualized in small physiological arteries, indicating restored normal venous circulation and successful fistula closure. Multimedia component. 1
